# Developmental robustness of the shoot apical meristem despite sex-chromosome dosage imbalance in *Silene latifolia*

**DOI:** 10.1186/s12870-026-09233-y

**Published:** 2026-06-11

**Authors:** Taiki Kobayashi, Kosuke Maekita, Yusuke Kazama

**Affiliations:** 1 Graduate School of Bioscience and Biotechnology, Fukui Prefectural University, 4-1-1 Kenjojima, Matsuoka, Eiheiji-cho, Japan; 2 Department of Bioscience and Biotechnology, Fukui Prefectural University, 4-1-1 Kenjojima, Matsuoka, Eiheiji-cho, Japan; 3 RIKEN Nishina Center, 2-1 Hirosawa, Wako, Saitama 351-0198 Japan

**Keywords:** Dioecy, *Silene latifolia*, Shoot apical meristem, *WUSCHEL*, Dosage compensation

## Abstract

**Background:**

Maintaining developmental stability despite sex-linked gene dosage imbalance represents a fundamental challenge for dioecious species. In the dioecious plant *Silene latifolia*, floral sexual dimorphism is associated with the sex-linked *WUSCHEL* (*SlWUS1*) and a *CLAVATA3*-like gene (*GSFY*) resulting in sex-specific differences in *WUS–CLV3* copy number. Because the *WUS–CLV3* feedback loop is a central regulator of stem cell homeostasis in the shoot apical meristem (SAM), such dosage imbalance would be expected to destabilise meristem maintenance.

**Results:**

We performed a quantitative analysis of SAM size in male and female *Silene latifolia* plants across vegetative developmental stages. Seedlings were grown in liquid half-strength Murashige and Skoog (½ MS) medium, and SAM morphology was examined using differential interference contrast microscopy. SAM diameter and area were subsequently measured using ImageJ. SAM size did not differ between male and female plants throughout vegetative development, despite differences in *WUS* copy number. This robustness persisted following exogenous application of the GSFY peptide (GSFYp) and cytokinin (CK), both of which significantly modulated SAM size and *WUS* expression. Expression analyses showed that the X-linked *SlWUS1* is weakly expressed in the SAM, whereas the autosomal *SlWUS2* exhibits high and sex-independent expression and responds similarly to peptide and CK signals in both sexes.

**Conclusions:**

These findings indicate that stem cell homeostasis in *S. latifolia* is primarily maintained by the autosomal *SlWUS2*, which buffers sex-linked dosage variation without relying on classical gene dosage compensation. Our results suggest the presence of a regulatory architecture that protects essential developmental processes from destabilisation during the evolution of dioecy.

**Supplementary Information:**

The online version contains supplementary material available at 10.1186/s12870-026-09233-y.

## Background

Angiosperms exhibit remarkable diversity in their sexual systems. In most angiosperm species, flowers possess both stamens and gynoecia and are therefore hermaphroditic. In contrast, only approximately 6% of angiosperms are dioecious, in which male flowers with stamens and female flowers with pistils occur on separate plants [1–4]. Dioecious plants are thought to have evolved from their hermaphroditic ancestors through two principal pathways: *via* gynodioecy, in which populations comprise female and hermaphroditic individuals, or *via* monoecy, in which male and female flowers are borne on the same individual [5–7]. The evolution of dioecy through gynodioecy involves at least two distinct mutations that cause male and female sterility. The first is a recessive mutation that suppresses the development of stamens or pistils in hermaphroditic plant populations, leading to the evolution of gynodioecy or androdioecy, respectively. The second is a dominant mutation that suppresses pistil or stamen development, resulting in the evolution of dioecy from gynodioecy or androdioecy, respectively [6, 8–10]. These “two-genes” or “two-mutations” models have been supported by numerous studies of dioecious plant [11–15]. Among these species, *Silene latifolia* has played a central role in advancing our understanding of sex determination and the evolution of sex chromosomes [16–18].

*Silene latifolia* is a dioecious plant in the family Caryophyllaceae that possesses XY-type sex chromosomes [19–21]. The morphological differences between male and female flowers of *S. latifolia* are controlled by two independent regions on the Y chromosome [22–24]. One region, termed the *stamen promoting factor* (*SPF*), promotes early stamen development in male flowers. Plants lacking this region develop asexual flowers [12, 25, 26]. The other region, termed *gynoecium suppression function on Y chromosome* (*GSFY*), suppresses early gynoecium development in male flowers. Plants lacking this region develop hermaphroditic flowers [12, 26, 27]. By analysing deleted regions of the Y chromosome in 11 hermaphroditic mutants, the gene responsible for *GSFY* was identified as an orthologue of *Arabidopsis thaliana CLV3 *[28].

*CLV3*, together with *WUS*, forms a feedback regulatory loop that controls stem cell homeostasis in the shoot apical meristem (SAM) and contributes to gynoecium formation [29–31]. The *WUS–CLV3* feedback system is also thought to play a critical role in gynoecium development in *S. latifolia *[32]. *SlWUS1* is located on the X chromosome but is absent from the Y chromosome [33, 34]. In addition, the genome of *S. latifolia* contains an autosomal paralogue of *SlWUS1*, termed *SlWUS2 *[33], as well as an autosomal paralogue of *GSFY*, termed *SlCLV3 *[35]. It has therefore been proposed that gynoecium development in *S. latifolia* is regulated by sex-specific differences in the copy number of these *WUS–CLV3* orthologous genes [15, 35].

Analysis of *WUS–CLV3* copy number in *S. latifolia* revealed that males possess three copies of *WUS* (*SlWUS1*; 1 copy and *SlWUS2*; 2 copies) and three copies of *CLV3* (*GSFY*; 1 copy and *SlCLV3*; 2 copies), whereas females possess four copies of *WUS* (*SlWUS1*; 2 copies and *SlWUS2*; 2 copies) and two copies of *CLV3* (*GSFY*; 0 copies and *SlCLV3*; 2 copies) [32, 35]. Given the importance of the *WUS*–*CLV3* feedback loop in regulating stem cell homeostasis, sex-specific differences in gene copy numbers are expected to affect floral meristem size. Indeed, female floral meristems are larger than those of males, particularly in the inner whorls [36, 37]. In addition, *SlWUS1* expression is significantly higher in female floral buds than in males [33], consistent with the observed sexual dimorphism in floral meristem size. These observations raise the possibility that SAM size may differ between the sexes. However, an earlier qualitative study reported no apparent sexual dimorphism in the SAM size [38]. This apparent discrepancy implies that SAM may be maintained through compensatory regulatory mechanisms that buffer differences in *WUS*/*CLV3* copy number between the sexes.

Here, we quantitatively examined sex-specific differences in SAM size in *S. latifolia* under normal conditions and following treatment with the GSFY peptide and cytokinin (CK), both of which influence SAM size and the expression of *WUS* genes [28, 39, 40]. Through these analyses, we investigated the regulatory mechanisms underlying SAM size control in the presence of differing *WUS–CLV3* copy numbers in *S. latifolia*.

## Methods

### Plant material

An inbred *S. latifolia* line (hereafter referred to as the K-line), maintained by repeated sib mating for 15 generations in our laboratory, was used in this study [20]. For SAM analysis, 20 seeds were placed in each 50 mL tube (WATSON, Kobe, Japan) containing 20 mL of half-strength liquid Murashige and Skoog medium (½ MS) [41] supplemented with 3% (w/v) sucrose and Gamborg’s B5 vitamins. The tubes were incubated on an RT-50 rotator (TAITEC, Saitama, Japan) at 23 °C under a 16 h light/8 h dark photoperiod for 14 days.

### Examination of SAM size

The incubated *S. latifolia* seedlings were classified into developmental stages based on the number of true leaves. Seedlings were fixed overnight at 4 °C in ethanol: acetic acid (3:1) and subsequently stored in 100% ethanol at 4 °C until further use. After washing with water, the seedlings were cleared using chloral hydrate (Nacalai Tesque, Kyoto, Japan). Samples were then imaged using differential interference contrast (DIC) microscopy with an LSM900 microscope (Carl Zeiss, Oberkochen, Germany) and scanning electron microscopy (SEM) with a FlexSEM 1000 II microscope (Hitachi High-Tech, Tokyo, Japan) equipped with a cooling stage maintained at − 20 °C. The area and diameter of SAM were measured using ImageJ software (version 1.54p, National Institutes of Health, USA) [42].　The SAM area and diameter are shown in Supplementary Fig. 1.

### DNA extraction and genomic PCR

Genomic DNA was extracted from the leaves collected from individual plants before fixation, and SAM observations were performed. The extracted DNA was subjected to PCR using the male-specific MS4 primer set [43] to determine plant sex. PCR amplification was performed for 35 cycles (98 °C for 10 s, 60 ℃ for 5 s, and 68 °C for 1 s) using KOD One polymerase (Toyobo, Osaka, Japan).

### Treatment with GSFY peptide and 6-benzyladenine (BA)

Following the established CLV3 peptide production protocol [44], a dodecapeptide corresponding to GSFY (GSFYp) was synthesised. Two proline residues were substituted with hydroxyproline residues to mimic post-translational modification.

Peptide treatment of the SAM was conducted as described previously [28]. Briefly, surface-sterilised *S. latifolia* seeds were placed in half-strength liquid MS medium containing 1% (w/v) sucrose, either with or without 0.1 µM GSFYp. Seeds were incubated at 4 °C for 3 days and then transferred to 23 °C under a 16 h light/8 h dark photoperiod for 14 days.

For the 6-benzyladenine (BA) treatment assay, surface-sterilised seeds were first incubated in half-strength liquid MS medium containing 1% (w/v) sucrose at 4 °C for 3 days, followed by incubation at 23 °C under a 16 h light/8 h dark photoperiod for 7 days. The seedlings were then transferred to fresh liquid MS medium containing 1% (w/v) sucrose with or without 4.44 µM BA and incubated for an additional 7 days.

For both GSFYp and BA treatments, incubations were performed in 50 mL tubes (20 seeds per tube) containing 10 mL of liquid medium on an RT-50 rotator (TAITEC). Following treatment, SAM morphology was analysed as described above.

### RNA extraction and quantitative RT-PCR

SAM tissues from male and female *S. latifolia* plants treated with GSFYp, BA, or left untreated were fixed overnight in ethanol: acetic acid (3:1) and subsequently transferred to 100% ethanol. RNA was extracted from the fixed SAM tissues using a NucleoSpin^®^ RNA Plant kit (Macherey-Nagel, Düren, Germany). Extracted RNA samples were treated with DNase (Thermo Fisher Scientific, Waltham, MA, USA), followed by cDNA synthesis using FastGene Scriptase II (Nippon Genetics, Toyama, Japan). Expression levels of *SlWUS1* and *SlWUS2* were quantified using real-time PCR. Amplification was performed for 55 cycles (95 °C for 10 s, the calculated annealing temperature for 10 s, and 72 °C for 10 s) using FastStart Essential DNA Green Master (Roche, Basel, Switzerland). The primer sets qSlWUS1_F/R, qSlWUS2_F/R, and qSlGAPDH_F/R were used. Primer sequences and annealing temperatures are listed in Supplementary Table 3.

### Statistical analysis

All statistical analyses were performed using R software (version 4.3.2). Differences in SAM diameter and area between male and female plants across developmental stages were evaluated using the Wilcoxon rank-sum test. For treatment experiments, differences among groups were analyzed using one-way analysis of variance (ANOVA) followed by Tukey’s honestly significant difference (HSD) test. Two-way ANOVA was additionally used to evaluate the effects of sex and treatment conditions where appropriate. *p* < 0.05 was considered statistically significant.

## Results

### No difference in SAM size between male and female *S. latifolia*

**Fig. 1:**
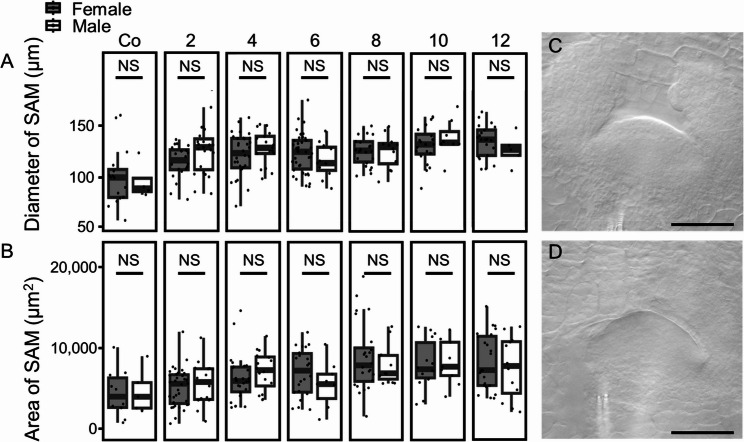
Comparison of shoot apical meristem (SAM) size between male and female Silene latifolia. **A**, **B** Quantitative analysis of SAM diameter (**A**) and SAM area (**B**) in male and female plants across seven developmental stages. Developmental stages were defined by the number of true leaves (Co, cotyledon stage; 2–12, number of true leaves). No significant differences (NS) were detected between sexes at any stage (Wilcoxon rank-sum test, *p* > 0.05). **C**, **D** Representative differential interference contrast (DIC) microscopy images of the SAM in a female (**C**) and a male (**D**) plant at the four-leaf stage. Scale bars = 50 μm

To determine whether sexual dimorphism exists in the SAM of *S. latifolia*, the sex of individual plants was first determined by PCR using the male-specific MS4 marker [43] (Supplementary Fig. 2). SAM morphology was subsequently analysed using DIC microscopy. SAM diameter was measured at seven developmental stages, ranging from the cotyledon stage to the twelve-leaf stage. At least four samples were analysed for each sex at each developmental stage. Quantitative analysis revealed no significant differences in SAM diameter between male and female plants at any of the developmental stages examined (Wilcoxon rank-sum test, *p* > 0.05) (Fig. 1A). Consistently, measurements of SAM area between leaf primordia also showed no significant sexual differences across all stages analysed (Wilcoxon rank-sum test, *p* > 0.05) (Fig. 1B). To further confirm these observations, SAM diameter was additionally measured using SEM microscopy. Consistent with the DIC microscopy results, no significant differences in SAM diameter were detected between male and female plants at any developmental stage (Supplementary Fig. 3). Taken together, these results indicate that the SAM size does not differ between the sexes in *S. latifolia*.

### No sexual difference in SAM size after CK or peptide treatment

**Fig. 2:**
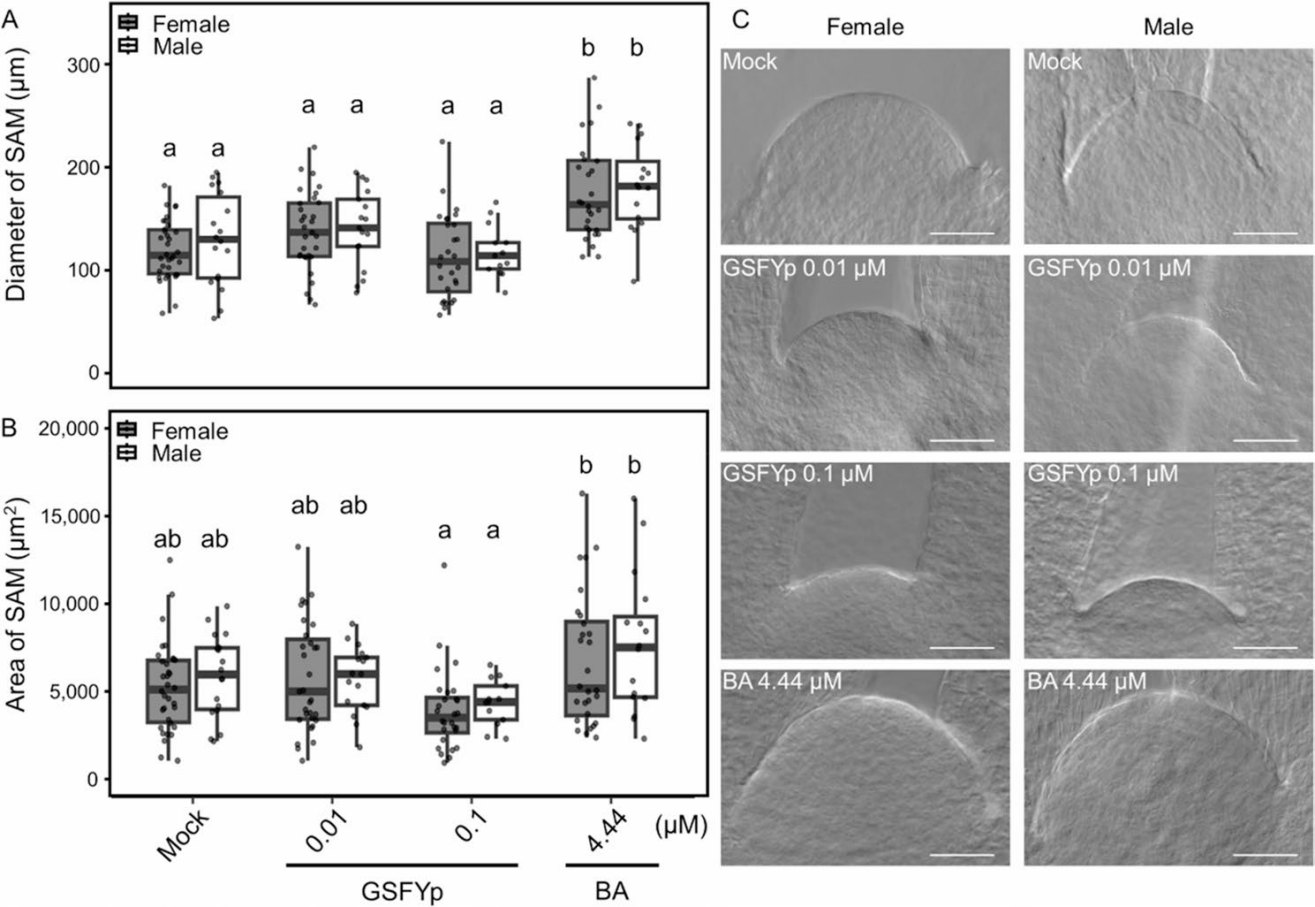
Robustness of SAM size in male and female *Silene latifolia* under peptide and cytokinin treatments. **A**, **B** Quantitative comparison of SAM diameter (**A**) and SAM area (**B**) between male and female plants under different treatment conditions. Seedlings were treated with 0.01 µM GSFYp, 0.1 µM GSFYp, or 4.44 µM 6-benzyladenine (BA). Different letters (a, ab, b) indicate statistically significant differences among treatment groups (one-way ANOVA followed by Tukey’s HSD test, *p* < 0.05). No significant differences were observed between sexes within any single treatment group. **C** Representative DIC microscopy images of SAMs from female and male seedlings. From top to bottom: Mock, 0.01 µM GSFYp, 0.1 µM GSFYp, and 4.44 µM BA treatment. Scale bars = 50 μm

Sex-dependent differences in the copy numbers of *GSFY* and *SlWUS1 *[35] suggest that exogenous GSFY peptide signalling induces sex differences in SAM size, thereby revealing whether the observed similarity between sexes is stable under external perturbation. To test this hypothesis, we applied a synthetic GSFY peptide (GSFYp), which has previously been shown to reduce SAM size in a manner similar to the CLV3 peptide [28, 45, 46]. Application of 0.1 µM GSFYp reduced the SAM area relative to the Mock treatment in male and female plants; however, this difference was not statistically significant (ANOVA with Tukey’s HSD test; Fig. 2A). No significant difference in the SAM area was detected between GSFYp-treated male and female plants, and both sexes were assigned to the same statistical group (Fig. 2A). Measurements of SAM diameter produced comparable results, with no sex differences observed following GSFYp treatment (ANOVA with Tukey’s HSD test; Fig. 2B).

Cytokinin (CK) treatment is also known to influence SAM size by affecting *WUS* expression and protein stability [39, 40]. To determine whether CK responses differ between sexes, the synthetic CK 6-benzylaminopurine (BA) was applied. Comparison of BA-treated male and female plants revealed no significant differences in SAM diameter, with both sexes belonging to the same statistical group (ANOVA with Tukey’s HSD test, *p* < 0.05; Fig. 2A). Similarly, no significant sexual differences were detected in SAM area following BA treatment (Fig. 2B; Supplementary Table 1).

Although SAM size varied among treatment conditions (Mock, GSFYp, and BA), statistically significant differences were mainly observed between BA and certain GSFYp treatments, whereas differences among GSFYp and Mock conditions were not consistent. No significant differences were observed between the male and female plants within each treatment group (Supplementary Table 2). These results indicate that the similarity in SAM size between the sexes is maintained even after external stimulation.

### Expression analysis of *SlWUS1* and *SlWUS2* in the SAM

**Fig. 3:**
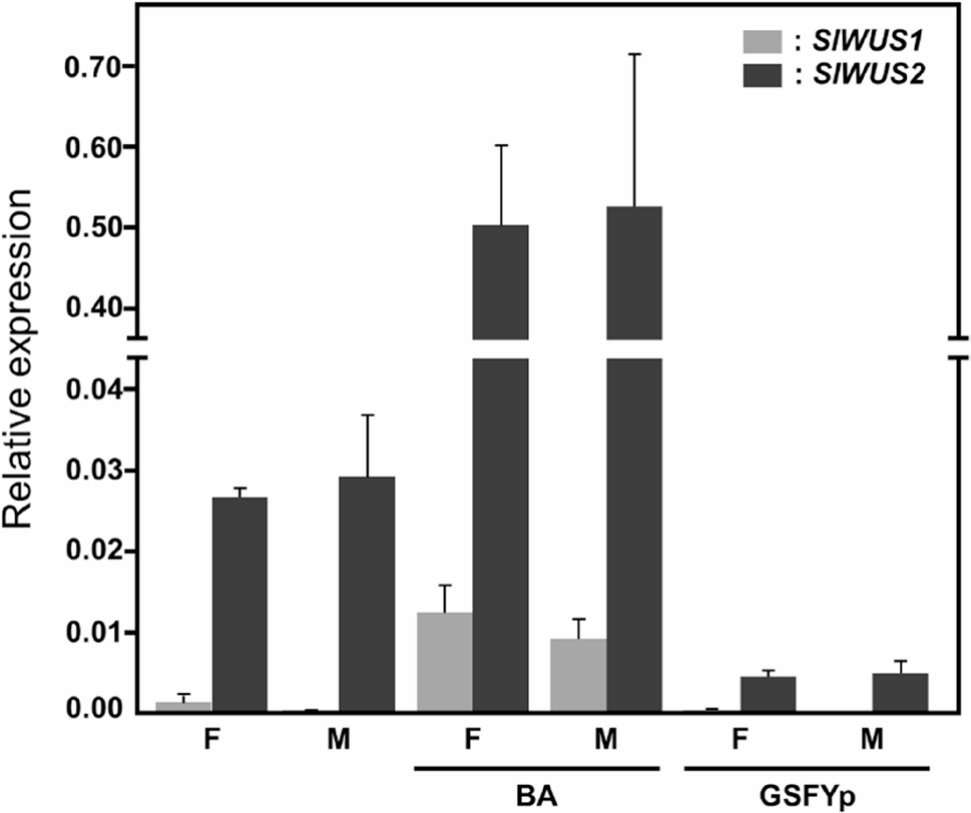
Expression analysis of *SlWUS1* and *SlWUS2* in the shoot apical meristem (SAM). Relative expression levels of *SlWUS1* and *SlWUS2* were determined by qRT-PCR in the SAMs of female (F) and male (M) plants under untreated (Mock), 6-benzyladenine (BA), or GSFY peptide (GSFYp) conditions. *SlGAPDH* was used as the internal reference gene. Although both *SlWUS1* and *SlWUS2* were expressed in the SAM, *SlWUS2* exhibited substantially higher expression than *SlWUS1* across all groups. No significant differences in *SlWUS2* expression were detected between male and female plants within each treatment group (Student’s t-test, *p* > 0.05). The error bars represent the standard deviation of three biological replicates

*WUS* is a key transcription factor involved in maintaining stem cell identity in the SAM [47–50]. Induction of the peptide gene *CLV3* represses *WUS* expression [51], whereas CK treatment promotes *WUS* transcription [52]. Therefore, changes in *WUS* expression provide a useful indicator of SAM responses to peptide and CK signalling. To assess the effects of GSFYp and CK treatments on the *WUS* orthologues in *S. latifolia* (*SlWUS1* and *SlWUS2*), their expression levels were quantified in SAM tissues from male and female plants. Expression of *SlWUS1* was significantly reduced following GSFYp treatment and significantly increased following BA treatment (Student’s t-test, *p* < 0.001; Fig. 3 and Supplementary Fig. 4). Similarly, expression of *SlWUS2* was significantly decreased by GSFYp treatment and increased by BA treatment (Student’s t-test, *p* < 0.001; Fig. 3).

Under Mock conditions, *SlWUS1* expression differed significantly between males and females (Student’s t-test, *p* < 0.001), reflecting its sex-linked copy number. However, overall *SlWUS1* expression levels were very low or undetectable. In contrast, *SlWUS2* expression was high and comparable between sexes. Taken together, these results suggest that the autosomal *SlWUS2* may play a predominant role in regulating SAM size, thereby contributing to the absence of sexual dimorphism in this trait.

## Discussion

*WUS* activity plays a central role in maintaining stem cell identity in the SAM, and elevated *WUS* function is generally associated with prolonged meristematic activity and delayed organ initiation[53–56]. In *Silene latifolia*, the *WUS* orthologue *SlWUS1* is located on the X chromosome, whereas *SlWUS2* is located on an autosome [33]. If *SlWUS1* and *SlWUS2* contributed equally to SAM function, sex-specific differences in *WUS* copy number would be expected to result in differences in SAM size or developmental behaviour. However, our quantitative analyses revealed no differences in SAM size between male and female plants across developmental stages and experimental conditions. These findings are consistent with the earlier qualitative observations by Grant et al. (1994) and extend them by providing quantitative and molecular evidence that SAM size is maintained at similar levels in both sexes. Notably, the absence of sexual differences in SAM size was observed despite differences in *SlWUS* copy number between males and females, indicating that SAM size is tightly regulated in a sex-independent manner in *S. latifolia*.

The best-characterised mechanism that buffers the phenotypic effects of sex-dependent copy-number differences is gene dosage compensation (GDC), in which gene expression levels are equalised between sexes [57, 58]. The GDC system has previously been reported in *S. latifolia*, where many X-linked genes corresponding to transcriptionally silenced Y-linked genes exhibit upregulated expression from the X chromosome [59]. Rapid GDC responses of X-linked genes to the absence of Y-linked counterparts have also been demonstrated using heavy-ion-induced Y-deletion mutants [60]. However, GDC has not been observed for *SlWUS1 *[33]. Consistent with this, our expression analysis confirmed that *SlWUS1* expression in SAM tissues was higher in females than in males (Fig. 3). Nevertheless, the autosomal *SlWUS2* exhibited substantially higher expression levels than *SlWUS1*, effectively masking the sex-dependent difference in *SlWUS1* expression. These results suggest that SAM size is primarily regulated by the autosomal *SlWUS2*, rather than through GDC of the X-linked *SlWUS1*. This regulatory architecture therefore represents a previously uncharacterised mechanism that buffers sex-specific differences in a key developmental trait, operating independently of canonical GDC pathways.

Consistent with observations reported in *A. thaliana *[39, 40], BA treatment resulted in SAM enlargement in *S. latifolia*, accompanied by increased expression of both *SlWUS1* and *SlWUS2*, whereas treatment with GSFYp caused SAM reduction together with decreased expression of these genes (Fig. 3). These results indicate that *SlWUS1* and *SlWUS2* expression in *S. latifolia* responds to SAM-modulating signals in a manner consistent with the conserved role of *WUS* in meristem maintenance. In *A. thaliana*, *WUS* expression is positively regulated by CK signalling and contributes to the maintenance of SAM size and stem cell activity [39, 40, 61]. CK signalling activates *WUS* transcription through type-B *ARABIDOPSIS RESPONSE REGULATORS* (*ARR1*, *ARR10*, and *ARR12*) [52, 62], and CK also contributes to the stabilisation of the WUS protein [40, 63]. Conversely, the CLV3 peptide, secreted from the central zone of the SAM, functions as a negative regulator of stem cell number [64, 65]. The CLV3 peptide is perceived by the receptor kinase *CLAVATA1*, leading to repression of both the expression level of *WUS *[29, 66–68]. Consistently, exogenous application or inducible expression of *CLV3* suppresses *WUS* expression [51, 69]. The conserved responses of *SlWUS1* and *SlWUS2* to BA and GSFYp treatments therefore suggest that the *cis*- and *trans-*regulatory mechanisms underlying hormonal responsiveness are likely conserved between these two genes.

In contrast, the basal expression levels of *SlWUS1* and *SlWUS2*, which appear to buffer the phenotypic consequences of sex-dependent copy-number differences, may be determined by distinct chromatin states. In *A. thaliana*, the *WUS* promoter is subject to multiple histone modifications that regulate transcriptional activity [52, 70–72]. However, chromatin dynamics within the SAM of *S. latifolia* remain largely unexplored. Further analyses will therefore be required to clarify the epigenetic mechanisms underlying the differential regulation of *SlWUS1* and *SlWUS2* in this species.

## Conclusion

Quantitative analyses revealed no differences in SAM size between male and female in *S. latifolia*, despite sex-specific differences in *SlWUS1* copy number. These findings suggest that SAM size is maintained through a sex-independent regulatory mechanism. The robustness of SAM size maintenance appears to arise from differential expression of the paralogous genes *SlWUS1* and *SlWUS2*. The autosomal *SlWUS2* is highly expressed in the SAM and appears to play a predominant role in regulating its size, whereas the X-linked *SlWUS1* is only weakly expressed. Consequently, sex-biased differences in *SlWUS1* copy number do not translate into differences in SAM size. These findings suggest that functional divergence in gene expression between paralogous genes can buffer sex-specific copy-number differences, thereby preserving developmental stability without invoking classical GCD. At the same time, these observations do not exclude the possibility that *SlWUS1* retains functional roles in other developmental contexts or tissues, which may explain its evolutionary maintenance despite its limited contribution to SAM regulation.

## Supplementary Information

Supplementary Material 1.

## Data Availability

All data generated or analysed during this study are included in this published article and its supplementary information files.

## Abbreviations

ARR: ARABIDOPSIS RESPONSE REGULATORS
BA: 6-benzyladenine
*CLV3*: *CLAVATA3*
GDC: Gene Dosage Compensation
Df: Degrees of freedom
DIC: Differential Interference Contrast
*GSFY*: *Gynoecium Suppression Function on the Y chromosome*
MS: Murashige and Skoog
qRT-PCR: Quantitative Real-Time Polymerase Chain Reaction
SAM: Shoot Apical Meristem
SD: Standard Deviation
SEM: Scanning Electron Microscopy
*SPF*: *Stamen Promoting Factor*
*WUS*: *WUSCHEL*
