# Supplementary material for: Developmental robustness of the shoot apical meristem despite sex-chromosome dosage imbalance in Silene latifolia
Source: BMC Plant Biol. 2026 Jun 11;26:1403. doi: 10.1186/s12870-026-09233-y (PMC13479931; doi:10.1186/s12870-026-09233-y)
Supplement: Supplementary file 1 [file 12870_2026_9233_MOESM1_ESM.pdf]

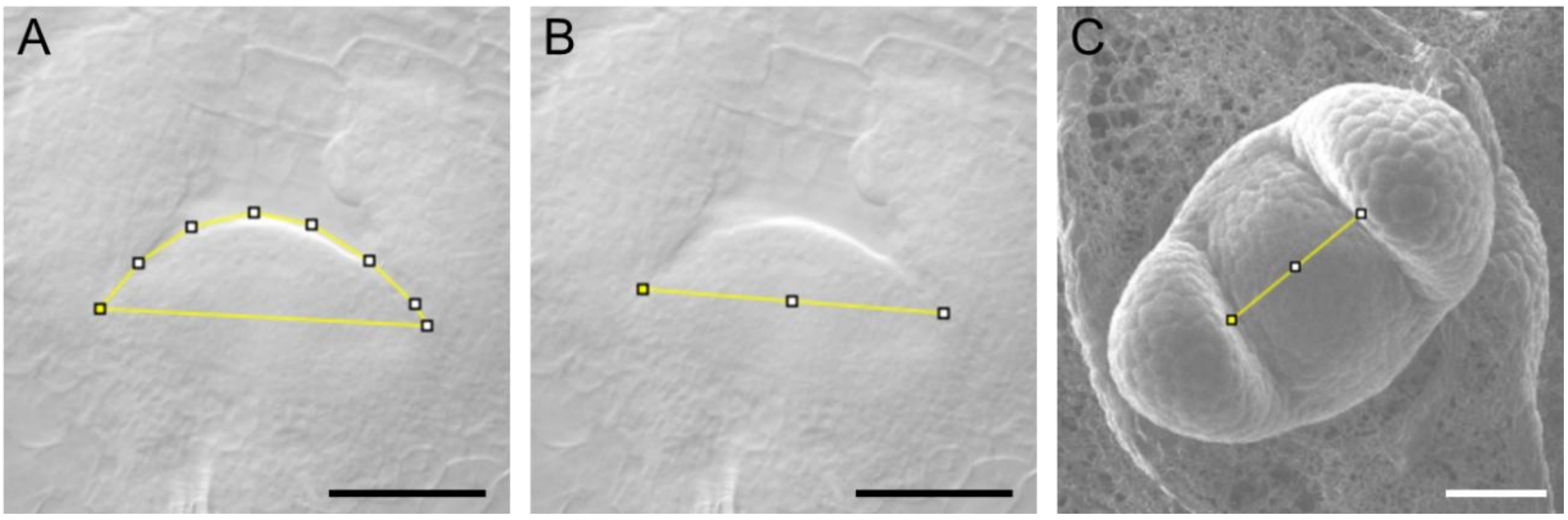


**Supplementary Fig. 1 Area and diameter measurements of SAMs.**

Representative images showing SAM sizes measured using ImageJ. (A) Measurement of the SAM area and (B) diameter in a DIC image. (C) Measurement of the SAM diameter in a SEM image. These images are representative examples of the images reused from the main figures. The lines drawn in ImageJ indicate the positions used for measurement. Bars = 50 μm.

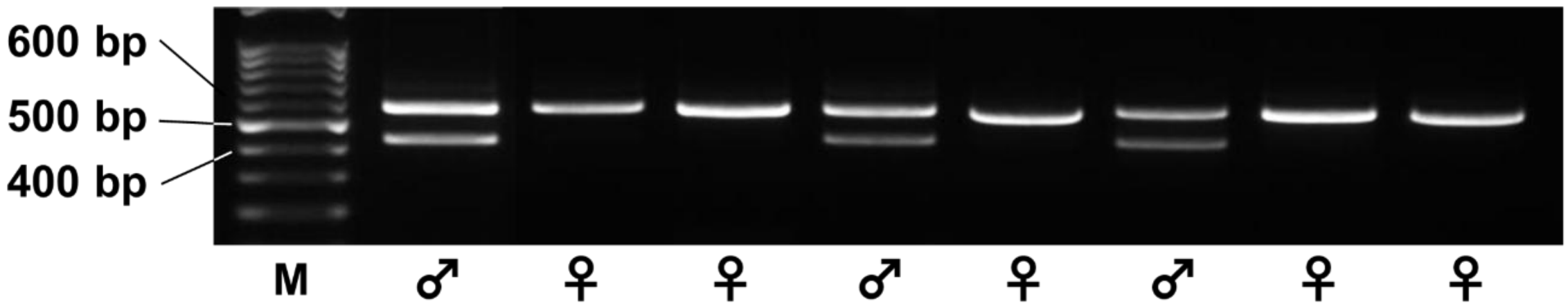


**Supplementary Fig. 2 Sequence tagged site PCR analyses of male and female individuals.**

PCR amplification was performed using genomic DNA from Silene latifolia seedlings. Fragments were amplified with the MS4 primer set41. Males have two bands; females have one band. Lane M contains SMOBIO® DM2300 using as the molecular marker.

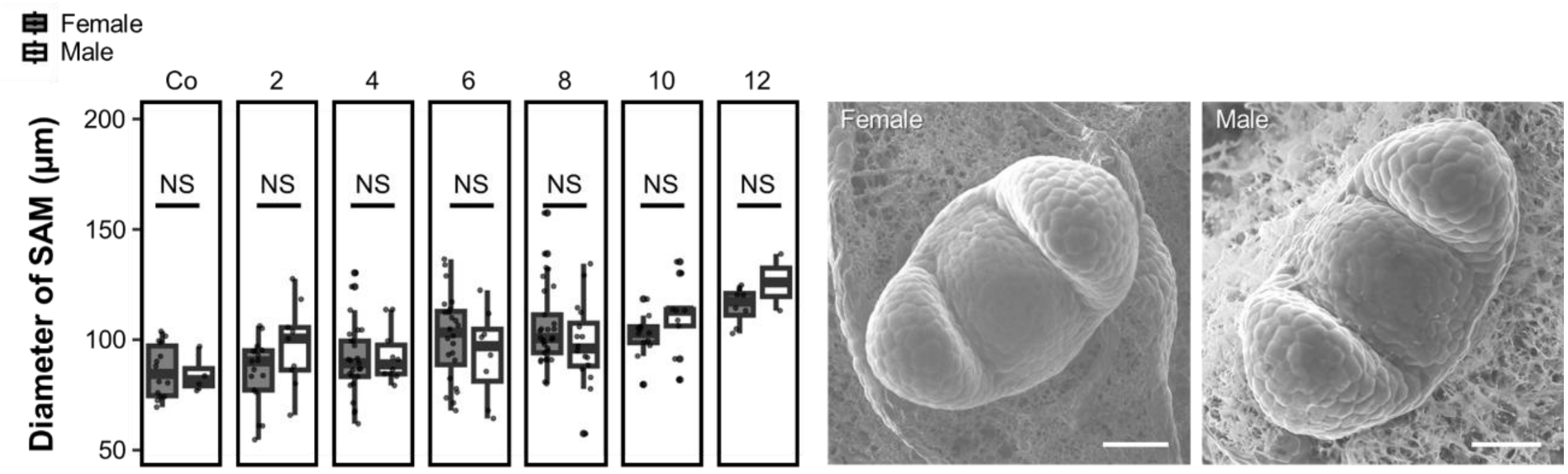


**Supplementary Fig. 3 Observation of Male and Female SAMs diameter at SEM.**

To compare the diameter of male and female SAMs in Silene latifolia. SAMs were classified by stage based on the number of leaves. The results showed no statistically significant difference in the diameter of male and female SAMs at any stage. Co; Cotyledon. 2~10; number of leaves

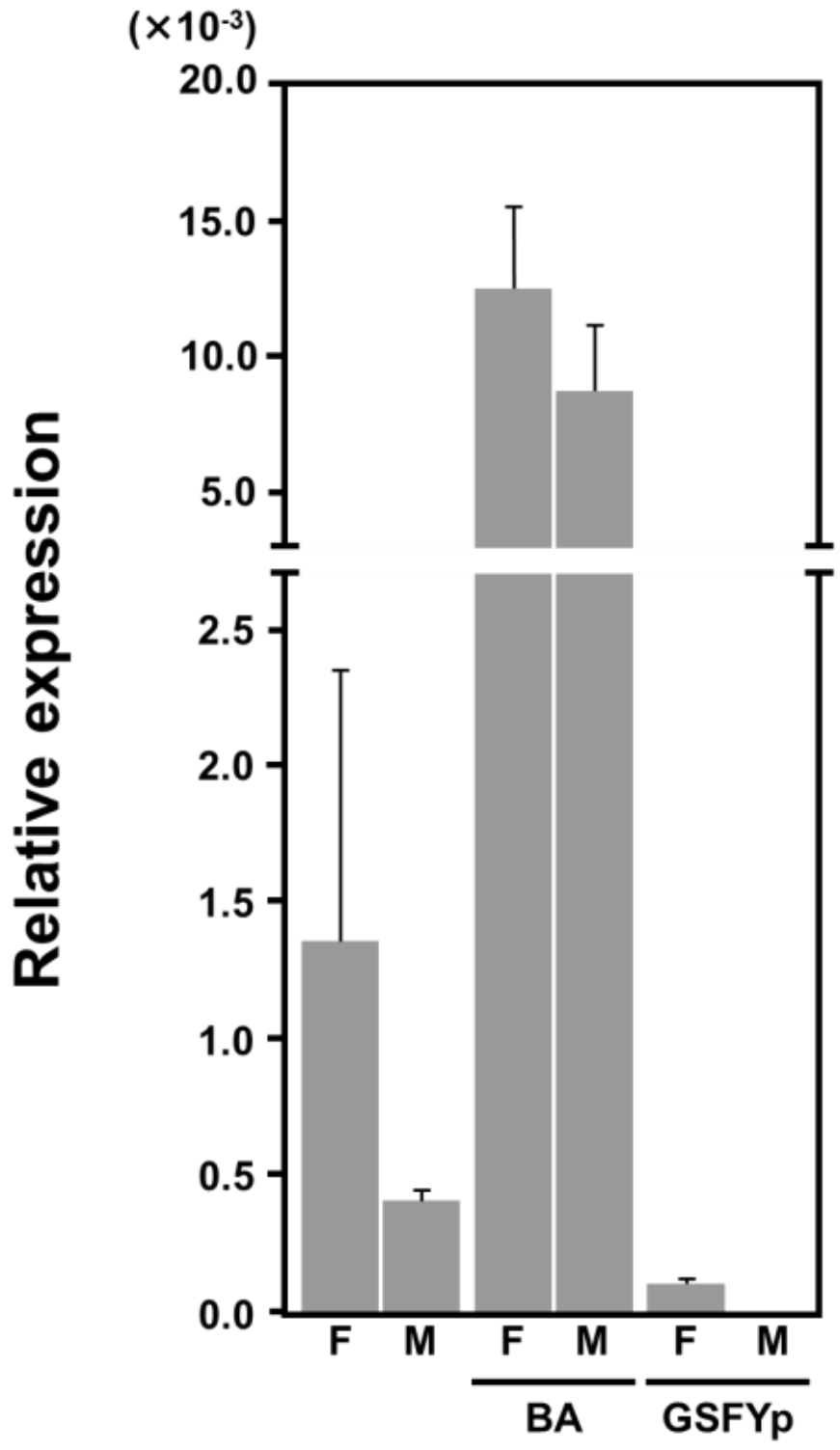


**Supplementary Fig. 4 Expression analysis of *SlWUS1* in the SAMs with an adjusted scale.**

Relative expression levels of *SlWUS1* in the SAMs of female (F) and male (M) plants determined using qRT-PCR under untreated (Mock), 6-benzyladenine (BA), or GSFY peptide (GSFYp) conditions. To improve the visualisation of the low *SlWUS1* expression levels, the y-axis scale was adjusted compared to Fig. 3. *SlGAPDH* was used as an internal reference gene. The error bars represent the standard deviation of three biological replicates.

**Supplementary Table 1.** Effects of GSFYp and BA treatments on the area and diameter of the SAM.

| | Line | Diameter mean ± SD (μm) | Area mean ± SD ($μm^2$) |
|---|---|---|---|
| Female | Mock | 119 ± 28.7 (a) | 5232.8 ± 2535.9 (ab) |
| | GSFYp_0.01 μM | 137 ± 38.1 (a) | 5820.7 ± 3130.8 (ab) |
| | GSFYp_0.1 μM | 113 ± 41.1 (a) | 3894.1 ± 2321.0 (a) |
| | BA_4.44 μM | 180 ± 55.3 (b) | 6743.4 ± 3787.7 (b) |
| Male | Mock | 129 ± 45.2 (a) | 5861.0 ± 2382.3 (ab) |
| | GSFYp_0.01 μM | 142 ± 37.5 (a) | 5669.4 ± 1906.3 (ab) |
| | GSFYp_0.1 μM | 118 ± 25.5 (a) | 4353.0 ± 1315.9 (a) |
| | BA_4.44 μM | 181 ± 42.4 (b) | 7687.3 ± 3986.9 (b) |

SD: Standard Deviation.

**Supplementary Table 2.** Results of two-way ANOVA testing the effects of line, sex, and their interaction on SAM diameter and area under GSFYp and BA treatments.

| | Diameter | | | Area | | |
|---|---|---|---|---|---|---|
| | Df | F value | *p* value | Df | F value | *p* value |
| Line | 3 | 22.98 | $1.30E^{-12}$ | 3 | 8.1042 | $4.35E^{-05}$ |
| Sex | 1 | 1.615 | 0.205 | 1 | 1.0987 | 0.296 |
| Line:Sex | 3 | 0.223 | 0.88 | 3 | 0.2769 | 0.842 |

Df: Degrees of freedom.

**Supplementary Table 3.** Primers used in this study.

| Primer | sequence | Tm (°C) |
|---|---|---|
| qSlWUS1_F | 5'- CTTACTCCCTCTAACACTACTACAAGTTC -3' | 59 |
| qSlWUS1_R | 5'- CCCCAGGATTATTGTTACCG -3' | |
| qSlWUS2_F | 5'- TTCAAGCTCATCAGCTGG -3' | 58 |
| qSlWUS2_R | 5'- CTCCTCCTGATATTGAACAGTC -3' | |
| qSlGAPDH_F | 5'- TGCTGACGTTAAGCCTGTTG -3' | 61 |
| qSlGAPDH_R | 5'- ACCAAGTCTATGCCCATTGC -3' | |

Tm: temperature of melting.
